# Odour Detection Methods: Olfactometry and Chemical Sensors

**DOI:** 10.3390/s110505290

**Published:** 2011-05-16

**Authors:** Magda Brattoli, Gianluigi de Gennaro, Valentina de Pinto, Annamaria Demarinis Loiotile, Sara Lovascio, Michele Penza

**Affiliations:** 1 Department of Chemistry, University of Bari, via E.Orabona 4, 70126 Bari, Italy; E-Mails: m.brattoli@chimica.uniba.it (M.B.); valentina.depinto@yahoo.it (V.P.); annamaria.demarinis@uniba.it (A.D.L.); saralovascio@yahoo.it (S.L.); 2 Brindisi Technical Unit for Technologies of Materials, ENEA, Italian National Agency for New Technologies, Energy and Sustainable Economic Development, P.O. Box 51 Br-4, I-72100 Brindisi, Italy; E-Mail: michele.penza@enea.it

**Keywords:** odour detection, odour concentration, sensory methods, dynamic olfactometry, electronic nose, sensors, sampling methods, GC-O

## Abstract

The complexity of the odours issue arises from the sensory nature of smell. From the evolutionary point of view olfaction is one of the oldest senses, allowing for seeking food, recognizing danger or communication: human olfaction is a protective sense as it allows the detection of potential illnesses or infections by taking into account the odour pleasantness/unpleasantness. Odours are mixtures of light and small molecules that, coming in contact with various human sensory systems, also at very low concentrations in the inhaled air, are able to stimulate an anatomical response: the experienced perception is the odour. Odour assessment is a key point in some industrial production processes (*i.e.*, food, beverages, *etc*.) and it is acquiring steady importance in unusual technological fields (*i.e.*, indoor air quality); this issue mainly concerns the environmental impact of various industrial activities (*i.e.*, tanneries, refineries, slaughterhouses, distilleries, civil and industrial wastewater treatment plants, landfills and composting plants) as sources of olfactory nuisances, the top air pollution complaint. Although the human olfactory system is still regarded as the most important and effective “analytical instrument” for odour evaluation, the demand for more objective analytical methods, along with the discovery of materials with chemo-electronic properties, has boosted the development of sensor-based machine olfaction potentially imitating the biological system. This review examines the state of the art of both human and instrumental sensing currently used for the detection of odours. The olfactometric techniques employing a panel of trained experts are discussed and the strong and weak points of odour assessment through human detection are highlighted. The main features and the working principles of modern electronic noses (E-Noses) are then described, focusing on their better performances for environmental analysis. Odour emission monitoring carried out through both the techniques is finally reviewed in order to show the complementary responses of human and instrumental sensing.

## 1. Introduction

In the last decade great attention has been paid to the issue of air quality as it directly affects both the environmental and human health. Air pollution has mainly an anthropogenic source: everyday industrial and commercial activities introduce an enormous and various amount of chemicals into the ambient air. Currently, people’s awareness of the effects of anthropic activities on the environment rises from the sensorial perception: nowadays olfactory nuisances, coming from various livestock buildings and industrial activities, are at the top of the list of air pollution complaints [1–3].

An odour is a mixture of light and small molecules, also at very low concentrations in the inhaled air, that, upon coming in contact with the human sensory system, is able to stimulate an anatomical response: the experienced perception is the odour [4]. Chemicals transported by the inhaled air are trapped and dissolved into the olfactory epithelium, a small region of both nasal cavities where odorants stimulate an electrical response of the olfactory nerves: the olfactory signal is thus transmitted to the brain, where the final perceived odour results from a series of neural computations. Odours are recognized thanks to the memory effect of previous experienced smells, thus accounting for the high subjectivity of the odour perception [5,6].

The human sense of smell has often been regarded as the least refined of all the human senses and far inferior to that of other animals. In fact, Aristotle (384–322 BC) blames this lack of finesse on the ducts in the human nose and claims that people who have noses with narrower ducts have a keener sense of smell, but he cites no experimental evidence for this assertion (Aristotle in *Problemata XXXIII*, and in *De Sensu et Sensibili in Parva Naturalia*). Moreover, the Roman philosopher Lucretius (99–55 BC) focused on the shape of the particles as conveying the quality of the odour and speculated on human olfaction by considering the nature and role of the odorant particles (Lucretius in *De Rerum Natura*). Also, the sense of smell is intimately linked with our emotions and aesthetics, but, despite the importance of odour, there is a lack of a suitable vocabulary to describe odours with precision. This is recognised by Plato in *Timaeus*: “the varieties of smell have no name, but they are distinguished only as painful and pleasant”.

The sense of smell enables people to detect the presence of some chemicals in the ambient air: in the worst cases an odour is associated with a risk perception [7,8]; anyway, generally, it is the marker for a specific situation or activity. Due to its nature, olfaction is becoming a tool of straightforward importance in various fields, such as food and beverage quality assessment [4,9,10] or illness detection [11]; in addition odour is more and more often regarded as an environmental concern [12–17]: a complaint arises just from the personal sense of smell [18–20]. The closer and closer proximity of industrial plants and farms, very often source of bad odours, to residential zones, really limits the acceptability of such activities and leads to citizen’s complaints [1,3,21]. Furthermore, odours strongly affect people’s daily life and health, as, although they do not represent a risk for human health, smells could cause both physiological symptoms (respiratory problems, nausea, headache) and psychological stress [22–24].

The growing concern for human and environmental well being, along with the increasing air pollution complaints submitted to regulators and government bodies, has promoted the necessity for effective odour impact assessment and consequent odour emission regulation [21]. A careful investigation of the odours issue requires odorous air measurement by applying standardized scientific methods [1,2,25,26].

Instrumental approaches to the characterization of odorants are based on the evaluation of the odorous air chemical composition. First of all the odorous air needs to be collected for subsequent analysis: the traditional VOCs sampling methods, like adsorbers or metal canister and polymer bags, are taken into account. The sampling procedures ensure the sample integrity, preserve the odour originally associated to the sample, minimize losses and chemical-physical interaction between odorants and the sampler medium [27,28].

Gas Chromatography coupled with Mass Spectrometry (GC/MS) has been widely used to analyse air quality, in order to produce a list of substances involved and their concentration [29,30], but the main limit of this technique relies on the complexity of the odour: the perceived odour results from many volatile chemicals, often at concentration lower than the instrumental detection limit, that interact synergistically or additively according to unpredictable rules [1,2,4]. Furthermore GC/MS instrumentation is expensive and does not give information about human perception, thus not allowing a linear correlation between a quantified substance and an olfactory stimulus [31]. Nevertheless, to overcome these limits, some efforts have been done in order to study the behaviour of odourants in a mixture and the potential masking phenomena that may occur [32,33], and to assess a relationship between instrumental and olfactometric methods [34].

The most sensitive and broader range odour detector is undoubtedly the mammalian olfactory system, whose high complexity and efficiency derive from millions of years of evolutionary development. The limits of traditional instrumental techniques in the matter of odours has led to growing attention to odour measurement procedures relying on the use of the human nose as detector, in compliance with a scientific method [4,5,35]. As occurring in the trade industry (*i.e.*, food, beverages, perfumes, *etc*.) for many years, the sensory evaluation of smells by means of panels of sensory trained evaluators has been the main odour assessment and quantification tool: the so-called dynamic olfactometry is the standardized method used for determining the concentration of odours and evaluating odour complaints [36,37]. This methodology is based on the use of a dilution instrument, called olfactometer, which presents the odour sample diluted with odour-free air at precise ratios, to a panel of human assessors. The examiners are selected in compliance with a standardized procedure performed using reference gases; only assessors who meet predetermined repeatability and accuracy criteria are selected as panelists. The odour concentration, usually expressed in odour units (ou/m^3^) is numerically equal to the dilution factor necessary to reach the odour threshold, that is the minimum concentration perceived by 50% of population [37,38]. According to European standardization, 1 ou/m^3^ is defined as the amount of odourant that, when evaporated into 1 m^3^ of gas air at standard conditions, causes a physiological response from a panel (detection threshold) equivalent to that of *n*-butanol (reference gas) evaporated into 1 m^3^ of neutral gas [37]. The perception of odours is a logarithmic phenomenon [39]; for this reason, in this kind of measurements it is necessary taking into account that odour concentration is associated to odour intensity though a defined logarithmic relation. Using other sensorial methods, subjective parameters, such as the hedonic tone or the perceived odour strength, could be assessed [37].

An improvement in odour determination consists of a GC-MS coupled with olfactometric detection (GC-MS/O) [40]. The gas chromatographic separation of an odorous air sample could be useful for identifying specific odorant components: GC-MS/O, thus, allows a deeper comprehension of the odorant composition as concerns the compounds’ identification and quantification, offering the advantage of a partial correlation between the odorant chemical nature and the perceived smell [41,42]. This instrumental approach tries to solve the odour complexity issue, which is also the main reason for the careful procedures required for the sampling of odorous air. Anyway the odour detection remains linked to the human perception. Although the careful choice of panel members and the use of standard procedures for odorous sample collection and analysis allow one to obtain reliable and repeatable olfactometric measures, thus overcoming the subjectivity due to the human olfaction variability, increasing attention is being paid to the availability of more objective odour evaluation methods.

The discovery of materials with chemo-electronic properties has provided the opportunity for the development of artificial olfactory instruments mimicking the biological system [4,9,43,44]. In the last decade a large field of scientific research has been devoted to the development of electronic-noses (E-Noses), that are sensor-based machines olfaction capable of discrimination between a variety of simple and complex odours. Like human olfaction, E-Noses are based on “an array of electronic-chemical sensors with partial specificity to a wide range of odorants and an appropriate pattern recognition system” [45]. In contrast to the ideal gas sensors, which are required to be highly specific to a single chemical species, sensors for E-Nose need to give broadly tuned responses like the olfactory receptors in the human nose: in both cases the odour quality information and recognition is ensured by the entire pattern of responses across the sensors array, rather than the response of any one particular sensor. Furthermore, mimicking the data processing in the biological systems, the incoming chemo-electronic signals are processed through the use of data reduction techniques (PCA); in both human and electronic noses, the function of odour recognition is finally achieved by means of some form of associative memory for the storage and recall of the previously encountered odours. A wide variety of competing sensor technologies (conducting polymers, piezoelectric devices, electrochemical cells, metal oxide sensors [MOX] and metal-insulator semiconductor field effect transistors [MISFETs]) are currently available: independently of the considered device, sensor elements have to show fast, reproducible and reversible responses to odour samples [43,46].

This review focuses on the state of the art of both human and instrumental sensing currently employed for odour assessment. The main features and the working principles of dynamic olfactometry and modern E-Noses, as monitoring tools for environmental analysis, are described. Papers comparing the performances of both techniques are finally reviewed in order to show the complementary responses of human and instrumental detection.

## 2. Sampling Methods for Odour Compounds

Sampling is a critical phase of the measurement procedure and requires particular attention in order to avoid sample losses due to sorption on the container or line surfaces and to minimize these interferences. Sample contaminations can easily occur if unsuitable or unclean materials are used; furthermore samples inevitably degrade or alter over the time: the choice of sample containers materials, the method for collecting odour and the time allowed between sampling and analysis are the main critical points of the sampling procedure [28,47].

### Materials

Materials for odour containers and sampling lines must themselves be odourless, undergo minimal physical or chemical reactions with the air sample and have low permeability in order to minimize sample losses through diffusion and/or adsorption. Stainless steel, polytetrafluoroethylene (PTFE), tetrafluoroethylene hexafluoropropylene copolymer (Teflon™), polyvinylfluoride (Tedlar™), polyterephtalic ester copolymer (Nalophan NA™) and glass are considered appropriate materials for odour sampling [37,38]. Therefore, odorous air is usually collected in stainless steel containers, called canisters, polymer bags or on adsorbent materials [48].

### Sampling Devices

Canisters are pre-cleaned evacuated cylinders useful for air sampling. Passivated canisters represent suitable devices for volatile and apolar molecules [49], as suggested by the most used standardized procedure [50]. The principal advantages of their use are that the air sample is collected without any breakthrough and there is no degradation of the trapping materials. Canisters need to be carefully conditioned and pretreated to avoid contamination problems and require complex sampling apparatus. Moreover the container volume is limited to a few liters, unless greater amounts of air samples are collected by means of pressurization, and they are more expensive than polymer bags [51,52]. Canister sampling does not work for dynamic olfactometry; only polymer-based bags are suitable for this use.

Polymer bags are mostly used for the collection of odorous compounds. In particular, sampling bags of materials such as Tedlar™ or Nalophan™ are considered appropriate [37,38,53]. Several researchers have investigated the features of plastic bags in order to verify the existence of background emissions. Keener *et al.* [54] and Trabue *et al.* [55] have shown that Tedlar™ bags emit acetic acid and phenol, which might bias air samples collected for olfactory analysis. Moreover, they have demonstrated that recovery of malodorous compounds is dependent on the residence time in the Tedlar™, bag with longer residence times leading to lower recovery. Reported background values in commercially available bags without pre-cleaning are in the range of 20–60 ou/m^3^ in Tedlar™ [56], 30–100 ou/m^3^ in Nalophan™ [57] or 2–30 ou/m^3^ and 10–50 ou/m^3^ in Tedlar™ and Nalophan™, respectively [58]. In these studies the authors have reported that flushing the bags with non-odorous air and, in some cases coupled by heating, background levels are reduced to about 10 ou/m^3^. Laor *et al.* [59] have tested the odour background from new bags and the impact of sample storage in both Tedlar™ and Nalophan™ bags, focusing on odours emitted from municipal sewage, aeration basins, sludge, livestock manure and coffee. They have verified that the odour background from new non-flushed Tedlar™ and Nalophan™ bags (in which fresh air have been stored for 24 h) is as high as 75–317 ou/m^3^ for Tedlar™ or 36–43 ou/m^3^ for Nalophan™. For pre-flushed bags the background is reduced to 25–32 ou/m^3^ for Tedlar™ or 19–22 ou/m^3^ for Nalophan™. This suggests that although new modern measurement systems allow us to detect very low odour concentrations, special caution is needed before considering values in the range of several to low tens of ou/m^3^.

Odour bags are filled using a depression pump that works on the basis of the “lung” technique; the bag is placed inside a rigid container evacuated using a vacuum pump [37,38,53]. This method avoids contamination because there is no direct contact between the pump and the sample. In order to get representative and reproducible results, it is necessary to adapt the sampling technique to the types of odour sources. In general, when a gas sample is very concentrated and/or it is very hot and humid, it is necessary to use a dilution device for avoiding condensation risks.

When sampling is performed by canisters or bags, the reactivity among the different compounds could compromise air sample stability and cause artifacts. For this reason, it is necessary that samples should be analyzed as soon as possible after sampling in order to minimize sample losses, degradation or alteration. Cheremisinoff [60] asserts that samples are still useful as long as 48 h after collection. In most cases, efforts are made to assess samples within 24 h of collection. The European Standard EN 13725/2003 states that odour samples must be analyzed within 30 h from sampling [37].

Sampling on adsorbent materials, packed in an appropriate tube, represents a handier sampling method than canisters and bags because it allows one to sample a great volume of air reducing the analytes in a small cartridge. The critical point is the choice of adsorbents (usually porous polymers or activated carbon, graphitized carbon black and carbon molecular sieves) [51,61–63], that depends on the chemical features of the compounds to be sampled [52]. A combination of different adsorbents is preferred to sample a wide class of compounds without breakthrough problems [62]. The sampling on adsorbent materials can be applied in “active” or “passive” mode. In active sampling, a defined volume of sample air is pumped at a controlled flow-rate. Passive or diffusive sampling occurs by direct exposure to the atmosphere; the process is governed by the adsorption properties of sorbent and diffusion processes [64–66]. The passive method does not require bulky and expensive pumps, that must be regularly checked, hindering field sampling, and it costs less than the active one. Moreover, particular care, on the choice of sampling volume, has to be taken to avoid breakthrough problems [51,52]. However, the active modality allows a greater and more accurate sampling volume. For both procedures the compounds can be recovered through thermal desorption or liquid extraction [65].

### Sampling Auxiliary Devices

The sampling devices described in the previous section are used for odour concentration monitoring in ambient air or for punctual emissions. In case of areal emissions [67], auxiliary devices are employed, depending on source features. Areal sources can be distinguished as active or passive. The first ones are characterized by a measurable outward airflow (*i.e.*, biofilters with forced aeration) while the latter do not have a measurable airflow (*i.e.*, landfills, cumulus, tanks, *etc*.). In the case of areal sources, it is generally very difficult to cover the whole emission area during sampling; for this reason, representative sampling sites have to be established and it is necessary using particular auxiliary devices for collecting odorous samples [68]. The investigations are conducted using a hood or a wind tunnel, depending on the measurement conditions. According to German VDI 3475 Bl. 1 [69] and VDI 3477 [70] a static hood should be used for sample collection on active areal sources, selecting a portion of the area and convoying the odourous air into the stack placed over the hood. For passive areal sources, a wind tunnel is positioned over the emitting surface; a known neutral air flow is introduced into the device, simulating the action of wind on the liquid or solid surface [71,72]. Different papers have focused on the evaluation of the performance of the existing types of chambers, hoods and tunnels used to collect volatile materials samples under different operative conditions [73]. Hudson and Ayoko [28,72] have shown that estimates of odour emission rates are strongly influenced by the selection of sampling device. Comparison of emission rates derived from turbulent and essentially quiescent sampling devices confirms that the concentrations and emission rates provided by these devices are quite different. Moreover emission rates measured with these devices are subject to external influences, including ambient wind speed and direction and the permeability of the emitting surface [72]. For improving the performance of these devices and optimizing efficiency parameters, special sampling chamber extension and a sampling manifold with optimally distributed sampling orifices have been developed for the wind-tunnel sampling system [74] and a suitable sampling system has been designed for the simulation of specific odour emission rates from liquid area sources without outward flow [75].

## 3. Sensory Methods

Sensory measurements employ the human nose as the odour detector, relating directly to the properties of odours as experienced by humans. Sensory measurement techniques can be divided into two categories:

1. Quantitative measurements which couple the nose with some instrumentation;
2. Parametric measurements in which the nose is used without any other device.

### 3.1. Instrumental Sensory Measurement

#### Dynamic Olfactometry

Instrumental sensory measurements employ the human nose in conjunction with an instrument, called olfactometer, which dilutes the odour sample with odour-free air, according to precise ratios, in order to determine odour concentrations.

The variables which will affect olfactometric measurements [12] are:

- olfactometer design;
- test procedure;
- differing sensitivity of observers;
- data quality;
- measurement uncertainty.

*Olfactometer design*. The materials used in olfactometer construction should not cause sample contamination or alteration through adsorption/desorption. Low-adsorbency materials such as stainless steel, Teflon, Tedlar™ or glass are used and internal surface areas are minimized. Risks of contamination can be prevented also supplying neutral air between the successive presentations.

*Test procedure.* In the choice of the order of sample presentation to the panel, it is important to consider that a descending order can enhance the effects of adsorption/desorption, and moreover it could provoke olfactory adaptation in panelists, since a weak odour (highest dilution) is more difficult to detect after exposure to a strong odour (lower dilution). Nevertheless, when dilutions occurr in a stict order, this kind of presentation can affect the panel response, because panelists expect subsequent samples to be weaker or stronger. Among these problems, the effects due to the choice of a descending order are more relevant, so an ascending order presentation is preferred [12].

There are two standardized methods for the presentation of odour sample to the panel: forced choice and yes/no method [37,38,53]. In the forced choice method, two or more sniffing ports are used; the odour sample is presented at one port, and neutral air at the other port(s). In this case, the examiners have to compare the different presentations and to choose the port from which odour exits. In the yes/no method each examiner sniffs from a single port and communicates if an odour is detected or not. Odour samples diluted with neutral air or only neutral air can exit from the sniffing port.

Sampling odour mixtures at different dilutions are presented to a group of selected panelists for sniffing and their responses are recorded. Generally, the first mixture presented to an odour panel is diluted with a very large volume of air in order to be undetectable by the human nose. In subsequent presentations, the volume of diluent is decreased by a predetermined and constant factor. After having set the factor, it is possible to create a geometric progression of dilutions (for example power factor of two: 2^16^, 2^15^, 2^14^,…) useful to describe the logarithmic relation between odour intensity and concentration [39]. The process continues until each panelist positively detects an odour in the diluted mixture; at this stage the panelist has reached the detection threshold for that odour [37,38,53]. This threshold is calculated as the geometric mean between the dilution of the last negative answer and the dilution of the first positive answer. The geometric mean is preferred for taking into account the logarithmic relation between odour intensity and concentration [39]. Different measurement cycles are carried out and the final result is calculated as the geometric mean of the values obtained for single series, as mentioned before [76].

The concentration is expressed as the dilution required for achieving panel detection threshold. Mathematically, the concentration is expressed as [77]:

(1) $$C=\frac{V_{0}+V_{f}}{V_{0}}$$

where C is the odour concentration, V_0_ the volume of odorous sample and V_f_ the volume of odour-free air required to reach the threshold.

By analogy, for a dynamic olfactometer the concentration is given by:

(2) $$C=\frac{Q_{0}+Q_{f}}{Q_{0}}$$

where Q_0_ is the flow of odorous sample and Q_f_ the flow of odour-free air required to reach the threshold.

The concentrations may be expressed as threshold odour numbers (TON) or dilution to threshold (D/T) ratios. Although the concentrations are dimensionless, it is common to consider them as physical concentrations, and to express them as odour units per cubic meter (ou/m^3^) [77,78].

*Sensitivity of observers: panel selection.* Panelists are qualified examiners used as sensors in olfactometric analysis and their olfactive response (odour threshold) is the measured parameter for calculating odour concentrations. However, the sensitivity to odours is variable among different individuals, so panelists could indicate different odour concentrations for the same sample. This effect is minimized because the examiners are selected according to a standardized procedure in order to choose individuals with average olfactive sensitivity, who constitute a representative sample of human population [37,38,53,79]. The screening is usually performed using reference gases [37,38,53,79]. In particular, the most used reference gas is *n*-butanol and only assessors who meet predetermined repeatability and accuracy criteria for this gas are selected as panelists [37]:

- average *n*-butanol odour threshold in a range of 20–80 ppb (40 ppb represents the accepted odour threshold for *n*-butanol)
- antilog standard deviation of individual responses less than 2.3.

Panelists must be continuously screened and trained and they must observe a simple behaviour code [34,35,50], whose fundamental prescription is that panelists impaired by illness caused by a cold or other indispositions are excluded from measurements.

*Olfactometric data quality*. Olfactometric data quality can be estimated according to two sources of uncertainty: the panel referability to a standard and the coherence of panel responses. In order to ensure the referability, the laboratory performances are evaluated by accuracy and precision measures. The assumption is that the laboratory performance to the standard odourant can be transferred to all odours tested by the laboratory. An example of criteria applied to verify the laboratory performance is reported as follows [37]:

**-** A_od_ ≤ 0.217, where A_od_ indicates the laboratory accuracy;
- r ≤ 0.477 or 10^r^ ≤ 3.0, where r indicates the laboratory precision, meaning that the difference between the results from any two consecutive measurements will not be larger than a factor three (3.0) for 95% of the cases.

The coherence of panel results can be estimated according to a validation procedure that permits one to exclude panel members who give invalid responses. An example of this type of procedures is represented by “retrospective screening” [37], based on the valuation of ΔZ parameter, calculated for each individual panel response as the ratio between the individual threshold value Z_ITE_ and the geometric mean of all individual threshold values *Z̄* *_ITE_* obtained during a measurement sequence:

(3) $$\text{If}\;{\text{Z}}_{\text{ITE}}\geq {\bar{Z}}_{\text{ITE}}\;\text{then}\;\Delta \text{Z}={\text{Z}}_{\text{ITE}}/{\bar{Z}}_{\text{ITE}}$$

(4) $$\text{If}\;{\text{Z}}_{\text{ITE}}<{\bar{Z}}_{\text{ITE}}\;\text{then}\;\Delta \text{Z}=-{\bar{Z}}_{\text{ITE}}/{\text{Z}}_{\text{ITE}}$$

This parameter must satisfy the following relation:

(5) −5≤ΔZ≤5

If one or more individual threshold values do not satisfy this criterion, then all responses given by the panel member with an inadequate ΔZ must be eliminated by the final result and the procedure is repeated until all data provided by panel member are consistent with the criterion. The ΔZ parameter indicates the coherence of panel members’ responses and puts in evidence the gaps eventually present compared to the mean. Moreover, so a measurement may be considered valid it is necessary that each panel member does not commit mistakes of more than 20% for the detection of neutral air [37].

In addition to these standardized procedures, different studies have focused on the determination of the analytical characteristics of the olfactometric method (reliability and robustness) with the purpose of determining the operating conditions influencing the final uncertainty associated with the measurements. In this field additional procedures for improving accuracy and repeatability of olfactometric measure, by optimization of panel selection [80], or by editing a quality control protocol based on interlaboratory comparison studies [81–83] have been evaluated. Moreover, panel repeatability tests have also been performed by presenting to panelists the same environmental odour sample or standard odorant multiple times during one test [84,85]. During these experiments, it has been shown that the time exposure affects panel response and that the optimal duration for the employment of analysts in a measure session is equal to two hours. By applying statistical methods, such as ANOVA, it has been demonstrated that olfactometric variance is mainly affected by within group variance compared to between group variance [84,85].

*Measure uncertainty.* Different attempts have been carried out for estimating a total uncertainty to assign to olfactometric measurements. As specified before, in this evaluation it is necessary to take into account the fact that the relation between odour intensity and odour concentration is logarithmic [39]. For this reason, the confidence interval is not symmetric around the average value [83,84]. It is possible to calculate an upper (UL) and a lower limit (LL) of the 95% confidence interval of the odour threshold, according to the following relations [86]:

(6) $$\text{lg}\;{\text{Z}}_{\text{UL}}=\text{M}+\text{t}\;\text{s}/\sqrt{N}$$

(7) $$\text{lg}\;{\text{Z}}_{\text{LL}}=\text{M}+\text{t}\;\text{s}/\sqrt{N}$$

where:

t: Student factor depending on f = L – W – 1
f: number of variances
L: total of measuring sequences
W: number of measuring sequences for series of measurements
N: number of panelists
M: arithmetic mean
s: standard deviation

#### Field Olfactometric Measurements

It would be ideal to carry out odour measurements directly at the odorous site, allowing continuous sampling of the odour without the need for storage. Unfortunately, this approach involves the need to isolate the panel of observers from the surrounding environment and to maintain them in an odour-free environment to prevent olfactory adaptation or fatigue. Usually *in situ* measurements can be performed using mobile laboratories even if their provision is much expensive. Instead of direct olfactometry, it is preferable to collect odour samples *in situ* and transfer them to an off-site odour laboratory for the assessment.

In 1958 the U.S. Public Health Service sponsored the development of an instrument and a procedure for field olfactometry (a technique only suitable for ambient odour concentration measurements). The first field olfactometer, called scentometer, is a hand-held device that allows one to evaluate odours on site. A field olfactometer creates a series of dilutions by mixing the odorous ambient air with odour-free (carbon-filtered) air. The U.S. Public Health Service method defines the dilution factor as Dilution to Threshold, D/T. The Dilution-to-Threshold ratio is a measure of the number of dilutions needed to make the odorous ambient air non detectable.

The advantages of scentometry are that it is economically attractive and readings are taken on site. Disadvantages include odour fatigue, because it is difficult not to expose the sniffer to the ambient environment (which is often odorous) before the scentometer is used, lack of dilution options and inability to rate sniffers against their ability to sense a known reference concentration. Because this test is conducted on site, some concern has been expressed regarding the ability of the sniffers to remain objective when they are seeing sources of odour emissions. These include rapid saturation of olfactory senses by some odorants, individual variation in sensitivity to different odours, fatigue as a result of adaptation, and changes in climatic variables (temperature, humidity, and wind speed) when measuring odours under field conditions, as well as effects of age, gender, health and personal habits on the sense of smell of individual panelists [87,88].

Two commercially available field olfactometers include the original scentometer, developed in the late 1950s, and the Nasal Ranger™, introduced to the market in 2002. These devices are used in studies regarding the evaluation of odour impact and have been compared with dynamic olfactometry or electronic noses [88], showing that Nasal Ranger field olfactometer is efficient at measuring livestock farm odour, and can provide consistent and accurate measuring results.

#### Hybrid Instrumentation: Gas Chromatography-Olfactometry (GC-O)

The opportunity of using sensory perception for the development of conventional instruments for chemical analysis has been investigated. Gas chromatography-olfactometry (GC-O) technique couples the traditional gas chromatographic analysis with sensory detection, in order to study complex mixtures of odorous compounds [40]. The GC-olfactometer consists of a traditional GC where a split column distributes the eluate between a conventional detector, such as a flame-ionization detector (FID) or a mass spectrometer (MS), and a sniffing port where a properly trained person or panel could detect the active odour species. All commercially available olfactometric ports are glass or PTFE cones, fitting the nose shape; the eluate is delivered through a dedicated transfer line which is heated to avoid the condensation of semivolatile analytes. In order to prevent the nasal mucous membrane drying, especially in long time analysis, auxiliary gas (humid air) is added to the eluate [89,90]. The sensory responses are recorded in an olfactogram: the eluate splitting occurs allowing the analytes to reach both human and instrumental detectors simultaneously, in order to compare the chromatogram with the olfactogram [89,91].

The combination of a mass spectrometer with an olfactometric detector is particularly advantageous as it allows the identification of odour-active compounds. Anyway, to avoid different retention times due to the different working pressure of the two detectors (a mass spectrometer and an olfactometer work under vacuum and atmospheric pressure conditions, respectively), particular attention is required for device assembling and in the choice of carrier and auxiliary gas flows [92].

Several methods have been developed to perform both qualitative and quantitative evaluation of the odour related to each analyte leaving the chromatographic column [89,93]. Dilution analysis methods, such as Charm (Combined Hedonic Aroma Response Measurement) Analysis [89,91,94,95] and AEDA (aroma extract dilution analysis) [89,91,96], are based on stepwise sample dilution, usually by a factor of two or three: each dilution is sniffed until no odour is detected, thus the highest dilution factor (FD) still allowing the odour perception is the odorant FD value. In the AEDA olfactogram each odorant is represented by a bar whose height is proportional to the odorant FD. In the Charm Analysis the beginning and the end of each odour perception is also taken into account, thus the olfactogram peaks combine the smell duration with the odour concentration [89,91]. Detection frequency methods use a group of assessors instead of one or two of them: the odour intensity of each compound is measured by means of the number of evaluators simultaneously detecting the odour at the sniffing port [97]. In direct intensity measurement methods, the intensity of the odour of the eluting compound is measured by means of different kinds of quantitative scales, thus single, time-averaged measurements, measurement registered after the elution of the analyte (posterior intensity evaluation method) or dynamic measurement (OSME, fingerspan method) are used [89,90,98]. The GC-O technique indicates the relevance of some chemicals in an odorant allowing the assessment of single compounds, but it does not provide information on their behaviour in a mixture [89].

The GC-O technique is widely used for the evaluation of food aromas [41,89,99–102], but its application in the environmental field is increasing, thus moving the odour emission assessment, from the solely olfactometric evaluation to the characterization of volatile components causing the odour nuisance. Odours emitted from animal production facilities have been often investigated by the GC-O approach in order to identify the compounds responsible of the primary odour impact and produce a deep screening of VOCs emitted in such activities by applying the GC-MS analysis [42,103–112]. It is often found that some compounds, due to their low odour threshold, can generate a high olfactory stimulus also at very low concentration; furthermore some odours are perceived at the olfactometric port also when the odorant compound is below the instrumental detection limit. Anyway the GC-O technique does not allow the evaluation of the additive and/or synergic effect of the single odorants in the true odour mixture, it limits its use to quantify the overall odour intensity [103–106].

Due to the high complexity of real odorous air samples, multidimensional GC is revealing a more powerful tool to allow a better livestock air resolution [42,108–111]. MDGC-O has also been employed to investigate the VOCs-odour-particular matter (PM) interactions, as suspended particulate is an important odour carrier [112].

### 3.2. Parametric Sensory Measurements

Parametric sensory measurements have the advantage of being quick to obtain at relatively low cost, as no particular equipment is required. Particular care has to be taken for interpretation of results due to the variation in odour perception, even for well-trained personnel [77]. Parameters which may be subjectively measured include odour character, odour intensity and hedonic tone.

*- Odour character*, often called odour quality, is a nominal scale of measurement. Odours can be characterized using a reference vocabulary with a standard list of descriptor terms [113].
- Perceived *odour intensity* is the relative strength of the odour above the recognition threshold (suprathreshold). Odour intensity is measured using several methods, including: descriptive category scales, magnitude estimation, and reference scales. There are several scales that usually employ 3–10 categories and panelists must assess the intensity of the sample according to the specified scale. The most common applied scale counts six categories [60,78,114] from no odour to very strong odour.

Systematic measurements on wastewater plants and waste treatment facilities and landfills have demonstrated that the intensity level of 3 (in a scale of six categories, it represents a distinct odour) is associated with an odorant concentration of approximately 4 ou/m^3^ [76].

Magnitude estimation is a procedure that compares the intensity of one odour with another odour. In this case, the assessor assigns an arbitrary value of intensity to the first odorant perceived and then attributes another value to the second sample on the basis of the first. This method is very difficult to apply to different types of odours, and is best suited for comparing similar odours [113].

The American Society for Testing and Materials recommends an intra-modal factory matching procedure with the use of an odour reference scale for the evaluation of suprathreshold odour intensity [115]. This standard presents two methods for referencing the intensity of ambient odours to a standard scale: dynamic-scale and static-scale. For dynamic scale dynamic olfactometry procedure is used, for static scale a test by a set of bottles with fixed dilutions of a standard odorant in a water solution [113] is performed.

- *Hedonic tone* defines the pleasantness and unpleasantness of an odorant. A method for determination of hedonic odour tone has been standardized [116]. Dilutions are presented through an olfactometer to the panelists. If the panelist detects an odour, the hedonic odour tone of the perceived concentration must be evaluated according to a category scale ranging from −4 (“extremely unpleasant”) through zero (“neither pleasant nor unpleasant”) to +4 (“extremely pleasant”) [76]. The influence of hedonic tone and intensity as suitable parameters for valuating odour impact and odour annoyance for residents living in the area surrounding industrial activities has been studied in several scientific works and taken into account in some government guidelines [17,21,117–119].

## 4. Electronic Noses and Olfaction Systems: Overview and Principles of Operation

Despite the importance of our perception of odour and flavour, there are problems in comparing different persons’ experience of smell and in quantifying odour. This need has created a desire for a more analytical approach to the quantitative measurement of odour. For this purpose the field of instrumental analyzers such as Electronic Noses (E-Noses) and Olfaction Systems (Machine Olfaction) has been developed in response to this desire [120,121].

The Electronic Nose is a device developed to reproduce the human olfactory system. It consists of three main parts:

- sampling system of odours to be analyzed;
- sensor system based on array of multiple sensing elements, or chemical sensors;
- data analysis and signal processing unit for feature extraction and significant information.

The response of the chemical sensors with partial selectivity is measured upon exposure to the sampled odour or multicomponent gas-mixture. The pattern based on the overall response of a sensor array defines a chemical fingerprint related to a given sampled odour. The recorded data of the sensors array response towards various odours can be usually processed by pattern recognition techniques (*i.e.*, artificial neural networks, multivariate statistical analysis) for their classification in order to identify odour and quantify the concentration. A proper set of features can be extracted from the recorded dataset to enhance the classification of odours without loss of significant information.

Despite the efforts to arrive at a universal device that can achieve fine discrimination of flavours, perfumes, smells, odours, analytes, and eventually replace the human nose, the E-Nose is not a chemical analyzer and thus must be trained for any specific application. However, this technical limitation of the E-Nose is combined to the potential ability of human odour sensing by increasing the number of performing individual sensors. This ability of the E-Nose to operate as biomimetic mammalian olfaction should be demonstrated yet. Nevertheless, there are strong drivers to apply E-Noses in the field of olfaction because alternatives, e.g., human test panels, either are not practicable or are too expensive and time-consuming/ In particular, E-Noses offer the advantages of real-time, *in situ* and remote control for olfactometric controls of air-emissions.

The term electronic nose first appeared in the literature in 1988 proposed by Gardner [122], it was discussed in a workshop on chemosensory techniques [123], and finally defined in 1994 [45]. Gopel *et al.* [124] in 1990 demonstrated the application of multicomponent analysis in chemical sensing for gas and odour detection. Ryan *et al.* [125] from NASA employed an E-Nose in the Space Shuttle to monitor air quality in the cabin. D’Amico *et al.* [126] demonstrated the monitoring of biological odour filtration in closed environments with olfactometry and electronic noses. Sberveglieri *et al.* [127] proposed a comparison of the performance of different features in sensor arrays for an E-Nose. Gardner *et al.* [128] proposed the development of a new olfaction system, called electronic Mucosa (e-Mucosa), based on advanced pattern recognition algorithms for space and time classification of odorants. Romain *et al.* [129] recently reviewed the use of metal oxide gas sensors for E-Nose environmental applications.

The detection of odours has been applied to many industrial applications. They include indoor air quality, health care, safety, security, environmental monitoring, quality control of beverage/food products and food processing, medical diagnosis, psychoanalysis, agriculture, pharmaceuticals, biomedicine, military applications, aerospace, detection of hazardous gases and chemical warfare agents.

### Chemical Sensors for E-Noses: Materials and Transducers

Chemical sensors for E-Nose applications need to be responsive to molecules in the gas phase. Many different types of gas sensors are available and some of them have been used in E-Noses at one time or another; however, nowadays, commercial instruments take into account two main types of gas sensors (metal oxide [MOX] and conducting polymer [CP] resistive sensors). Recent studies are focused on the evaluation of other types of solid-state gas sensors.

**Figure 1.**
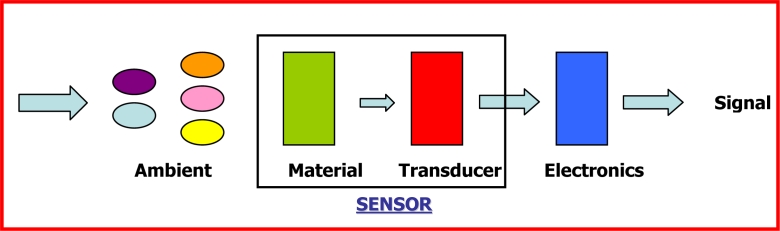
Scheme of a solid-state chemical sensor with gas-sensitive material, transducer and interface electronics.

Chemical sensors comprise an appropriate and chemically-sensitive material interfaced to a transducer, as shown in Figure 1. Hence, the solid-state sensors are essentially constituted by a chemically sensitive interface (sensitive material) and a transducer able to convert a chemical input (gas concentration or ions concentration) and/or physical input (temperature, pressure, acceleration, *etc*.) into an output, generally an electrical signal, by means of a conditioning and/or signal processing electronics [122]. The input magnitudes or measurands include chemical and/or biological magnitudes such as concentration and identity of unknown species in gaseous or liquid phase, other than physical general magnitudes such as temperature, pressure, speed, acceleration and force. A transduction process is realized by converting the input-event or measurand into an output electrical signal (analogue voltage or current, digital voltage) correlated to the measurand that generates it. The output electrical signal is properly conditioned, processed and stored for analysis.

Gas sensors, based on the chemical sensitivity of semiconducting metal oxides, are readily available commercially and have been more widely used to make arrays for odour measurement than any other single class of gas sensors. An in-depth overview on sensor materials for odour detection can be found in literature [130,131]. The most common sensor materials for odour measurements are listed in Table 1.

The classification of chemical sensors can be realized according to the transducer used. The various categories of solid-state chemical sensors are differentiated by the physical principle of the signal transduction by distinguishing the following transducers: conductometric (resistive), optical, electrochemical, mechanical/acoustic or ultrasonic, thermal and MOSFET. A detailed classification of the solid-state chemical sensors is given in Table 2, showing the principle of operation, the methods of sensor fabrication and some technical comments. Additional definitions and principles of operation have been reported in literature [132,133].

The measurements of the odour concentration by solid-state sensors implemented in the E-Nose should be standardized. Hence, the definition of the sensor parameters is essentially in this issue [132,133].

The main sensor parameters are:

- *Sensitivity:* is a measure of the magnitude of the output signal produced in response to a given input magnitude (perturbation/stimulus), or the ratio between two non-homogeneous magnitudes output signal/input measurand.
- *Response time:* indicates the time that the sensor signal takes to pass from 10% to 90% of its excursion to reach a new steady state, during the response dynamics.
- *Recovery time:* indicates the time that the sensor signal takes to pass from 90% to 10% of its excursion to reach a new steady state, during the recovery dynamics.
- *Resolution:* is the measure of the minimal variation of the input magnitude to which the sensor is able to response for a given signal-to-noise ratio, at a fixed working point.
- *Limit of Detection (LOD):* is the minimum gas concentration that a sensor is able to detect for a given signal-to-noise ratio.
- *Selectivity:* characterizes the capability of the sensor to distinguish a given input magnitude from another measurand belonging to a different class.
- *Drift:* is the attitude of sensor output signal caused not by an external input but by intrinsic reasons (sensor material, electronics) of the sensor.
- *Stability:* is the attitude of the sensor to keep constant in the time its metrological characteristics; in other words, its response in the time.
- *Repeatability:* is the attitude of the sensor output signal towards a given fixed input measurand in different repeated measurements.

### Applications of E-Noses for Environmental Analysis

The application sectors of E-Noses for odour monitoring are indicated as follows:

- measurement of odours produced by factories causing a public nuisance
- measurement and quantification of airborne odours from other sources: sewage farms, waste sites, agricultural activities, cattles, cars, *etc*.
- measurement of odours inside buildings that may arise from harmful building materials, faulty heating, ventilation systems
- measurement of odours in workplaces to preserve worker health.

Many multiparameter portable sensor-systems have been studied and exploited in field measurements for air quality control of toxic pollutants (NO_x_, CO, SO_2_, H_2_S) [134], greenhouse (CO_2_, CH_4_) [134,135], refrigerant gases [135], warfare agent simulants [136] with wireless functionalities [137] in urban areas [138] by using traditional (chemoresistive) [134,135,139,140] and innovative (SAW) [136,141] transducers.

Moreover, practical portable devices [142–145] have been developed for odour monitoring of landfill municipal sites and for odour quantification by a sensor array. In particular, Persaud *et al.* [143–145] used a single-point E-Nose instrument for continuous monitoring along the perimeter of a municipal landfill site by measuring methane and carbon dioxide as main components in a biogas produced by waste fermentation.

Additionally, the E-Nose has been applied in *in situ* measurements [146–153] for the identification of malodours sources [149], to control odour concentration emitted from a malodour agricultural site [147] and a compost hall [151], to monitor the odour emission from construction materials [150] and for the classification of fruity odours [153], including odour emissions from a biofiltration system in a pig farm building [152].

The new trends in the odour detection are strongly driven by nanotechnologies and nanomaterials [154–157]. Nanotechnology has attracted a lot of attention recently, particularly in the research and industrial communities. It offers many opportunities for advancing our ability to impact on day-life and environment. The ability to design, synthesize and manipulate specific materials at nanoscale lies at the very heart of the future promise of nanotechnology. Nanomaterials may have unique physical and chemical properties not found in their bulk counterparts, such as unusually large surface area to volume ratios or high interfacial reactivity. Such properties can be useful to develop new chemical capabilities arising from exciting new classes of nanomaterials (e.g., nanotubes, nanowires, nanocrystals, nanoparticles, *etc*.). Several studies concerning the use of nanomaterials as gas sensor materials have been reported in literature. Penza *et al.* [155] studied an array of four sensors based on carbon nanotube layers functionalized with metal catalysts for landfill gas monitoring applications. Lieber *et al.* [157] developed an individual silicon-nanowire to implement a field effect transistor (FET) functionalized with DNA and proteins for detection of biological and chemical species in the area of healthcare and life sciences. This device may be called a nanosensor. However, these nanosensors based on individual nanowires have been integrated by Cheng *et al.* [154] in an array of multiple sensing elements to implement a *nanoelectronic nose* based on hybrid nanowire/nanotubes and micromachining technology for sensitive gas discrimination. This nanoelectronic nose has great potential to detect and discriminate a wide variety of gases, including explosives, nerve agents and odours.

## 5. Olfactometry and E-Noses: Comparison and Integrated Approach

As concerning the different techniques applied to odour determination previously discussed, whose characteristics are summarized in Table 3, it was shown that no one of the described techniques is able alone to give exhaustive informations about the odorous emissions from different kinds of human activities that may cause olfactory nuisance. Therefore, a comparison and/or an integration of the olfactometry methods with the technologies of sensorial analysis is necessary to completely evaluate odour impact [158].

Several correlations can be observed between trends in the discrimination properties of the electronic nose and the human olfactory system [159]. Since E-Noses are not able to provide odour concentrations, many authors have focused their attention on the research of a correlation between olfactometric and sensorial results in order to realize a fast, portable and not very expensive device to carry out frequent odour measurements in case of complaints from the public or in presence of unstable odour compounds.

The dynamics of odour emissions from a pig barn have been investigated by olfactometry and using an electronic odour sensor. The sensor signals showed a good relation to the odour concentration and revealed a promising potential of electronic odour sensors to detect the dynamic and the level of odour concentrations [160].

On samples from pig and chicken slurry electronic nose measurements based on polypyrrole sensors have been evaluated against odour concentration measurements by the olfactometric technique; electronic nose sensitivity was found to be lower than the olfactometry one, showing the need to develop sensors to specific groups of compounds [161].

Thus, an electronic nose equipped with 14 gas sensors suitably selected for measuring odorous components from livestock farms has been developed. The responses of the sensors have been found to be in good agreement with the perceived odour intensities of a portable field olfactometer [88], and both the data sets, used to train an expert system for supporting odour management of livestock and poultry farm, have made possible to forecast the effectiveness of odour control efforts before those control means were applied [162].

An electronic nose based on conducting polymer sensors, has been widely applied in the analysis of odour samples from swine manure sources coupled with a NH_3_ and a H_2_S sensor [163,164] and as an alternative to sensory analysis for assessing the effectiveness of biofilters, showing good correlation with odour concentrations [165]; together with olfactometry and gas chromatography to analyze indoor air from swine finishing facilities, instead, the correlation between GC/MS analyses and E-Nose was found better than between E-Nose and olfactometry. This result suggested that human panelist responses may be based on detection of compounds that are not included in GC/MS quantification procedures and are not well detected by this electronic nose [166].

An electronic nose was used in an experimental farm to quantify the odour level inside the animal room and a good correlation was found with the olfactometric results on the same samples. E-Nose results showed an evolution of the odour with animal activities during the day and with their age [167].

Sohn *et al.* used an artificial neural network, trained by the data sets obtained with an electronic nose and dynamic dilution olfactometry, to predict the pig farm odour concentrations emanating from an effluent pond and to develop a confident, rapid, and cost-effective technique for odour measurement [168]; in addition they demonstrated the relationship between odour emission rates and pond loading rates on pig farm effluent ponds and the increased magnitude of emissions from a heavily loaded effluent pond [169].

As concerns livestock farms, they also employed olfactometry and electronic nose results to demonstrate the odour monitoring capability of a non-specific conducting polymer-based array for evaluating the performance of a biofiltration system installed at a commercial pig farm [152] and to develop an odour prediction model using PLS (Partial Least Squares) to investigate the relationship between odour concentrations inside the poultry shed and factors such as weather, bird age, ventilation rates and other variables associated with the broiler production cycle [170].

Agricultural sources can also be a source of complaints, so a device able to carry out field measurements is required. After application of cattle slurry to grassland, two olfactometers and two electronic noses were used, demonstrating the ability of both E-Noses to respond to the odour concentrations arising from cattle slurry applications at levels which would be similar to those from a range of agricultural sources [171].

Applying PCA (Principal Component Analysis) and then PLS regression, a good correlation between odour units and sensors data of E-Nose has been found in odour measurements from a rendering plant bio-filter inlet and outlet [172], and in investigations on the organic fraction of municipal solid waste [173], demonstrating that a correctly calibrated E-Nose could replace olfactometry as a tool for odour impact measurement.

On the other hand, studying samples from different sewage treatment works, a comparison between results of an electronic nose and dynamic olfactometry showed there is no universal relationship between the electronic nose responses and odour concentrations for sewage odours from a range of locations within different treatment works, but only for odour samples which are source/site specific [174,175]. The same result was obtained also on wastewater samples from different treatment works. [176].

Experimental studies have been carried out with an E-Nose to determine the detection limits of the selected sensors, using olfactometric measurements of odour detection threshold concentration, and the sensors capability of discriminating different odours in waste treatment plants. The sensors characterized by low detection limits for the considered odorants, also showed a good capability of discriminating these odorants from each other [177].

Moreover the use of a chemosensor system, calibrated with olfactometric data on a waste incineration plant, allowed continuous monitoring behind a charcoal filter and thus the identification of filter breakthrough [178].

Among the human activities that may generate problems related to unpleasant odour emissions, landfills represent one of the major causes of odour complaints [16]: they are difficult to monitor as they are characterized by a great variety of substances that may cause odour nuisance and then they require the use of more than one technique for odour determination.

For a complete characterization of odours at a landfill, Capelli *et al.* collected samples in different zones inside the plant, at the boundaries and at the receptors, and analyzed them with different techniques: olfactometry enabled a quantification of the landfill odour emissions, giving indicative values of sensory impacts; chemical analyses with GC-MS were useful to analyze odour composition, and electronic noses (two at the boundaries and one at the nearest receptor) were used as a management tool in order to monitor site changes or operational failures. This study has shown that even if the results of the three different odour characterization techniques do not necessarily correlate, each one contributes to solve the complexity of odour measurement in the environment [179].

Other comprehensive investigations on landfill areas used olfactometry with a dispersion modelling, odour patrol monitoring and an E-Nose [180]; dynamic olfactometry, field determination of odour perception points and electronic noses to create a calibration curve that allowed the translation of the global E-Nose response into odour concentration units that could be compared to a warning threshold concentration [181]. Another approach was carried out using results of olfactometric analysis as the input for a dispersion model and two electronic noses for continuous monitoring to determine the landfill odour impact on a specific receptor, and a very good correspondence of the electronic nose responses with the odour detections reported by the people living at the receptor and with the result of the odour dispersion modelling was found [182].

Some authors used data sets, obtained by evaluating odour samples with both an olfactometer and an electronic nose, to train artificial neural networks (ANN) and develop a function to convert the measurements of an electronic nose into odour concentrations. The odour concentrations measured with the olfactometer have been used as observed values, and the responses of the electronic nose as input variables [183]. Using this technique on composting plants, it was possible to get characteristic patterns only for different parts of the plant, but, for these parts a good similarity between the samples was shown [184].

For the estimation of odour disturbances from the biofilters for the treatment of emissions from a municipal solid waste organic fraction composting plant, dynamic olfactometry has been used to determine odour intensity and to verify the standards of odour disturbance in combination with an electronic nose. Once a correlation between the two methods was established, it was possible to carry out frequent quantitative determinations of the biofilter emissions by simply using the electronic nose, with consequent lower costs than dynamic olfactometry analysis [158].

The possibility of monitoring the time evolution of the odour concentration has also allowed the use of an electronic nose suitably calibrated by olfactory measurements to supply a warning signal when the compost odour is identified and exceeds a given threshold [151].

A problem that requires continuous monitoring, is the assessment of the presence of odours at a particular receptor, like a house whose owners often complain about the unpleasant odours originating from a nearby plant. For a composting plant the electronic nose response has been correlated with the odour concentration values measured by dynamic olfactometry in order to use the instrument for the continuous odour concentration measurement. Two electronic noses have been installed in the house and in the composting plant; in correspondence to the measurements during which the electronic nose inside the house detected the presence of odours from the composting plant, the olfactory classes recognized by both instruments coincided. Moreover, the electronic nose at the house detected the presence of odours from the composting plant at issue in correspondence of each odour perception of the house occupants [185].

An E-Nose was trained to analyze different gas samples of known olfactory quality at different odour concentration values, and then installed in two different periods at two receptors of a composting plant. Applying an appropriate data analysis, a high correlation index was found between true and predicted odour concentration values, thus demonstrating that an opportunely trained electronic nose and suitable data processing methods may represent a valid solution to the problem of having a system for continuously monitoring odours of environmental interest [186].

## 6. Conclusions

The increasing attention of the population to olfactory nuisances and the need to provide a reliable qualification and quantification of odours has led to the development of different odour measurement techniques. In particular, instrumental sensory methods and chemical sensors have been described, showing the advantages and disadvantages of each technique.

Although dynamic olfactometry represents the standardized objective method for the determination of odour concentration, it is affected by some limitations. First of all dynamic olfactometry provides point odour concentration data, however, it is not sufficient to evaluate completely a case of olfactory nuisance because it does not allow one to perform continuous and field measurements, useful for monitoring the industrial processes causing odour emissions. Moreover, dynamic olfactometry considers the whole odour mixture and do not discriminate the single chemical compounds and their contribution to the odour concentrations. Odour samples are difficult to store, because of their instability, and, therefore, require rapid time of analysis. Finally, as it is well-known, olfactometry is too time-consuming and quite expensive and moreover frequency and duration of analysis are limited.

On the other hand, electronic noses present lower analysis costs and quick results and they allow one to carry out continuous monitoring in the field nearby sources and receptors. After a training step, electronic noses are able to preview the class of an unknown sample and then to associate environmental odours to a specific source.

Since each technique satisfies only a part of the problems of odour monitoring, many authors have focused their attention on carrying out comparisons and integrations between olfactometry and E-Nose results. These applications show the opportunity of using more than one approach for describing and understanding olfactory nuisance cases as completely as possible.

## Figures and Tables

**Table 1.** Most used gas-sensitive materials for chemical sensors.

**Class of Materials**	**Sensor Materials**	**Technology**
Thin-film metal oxides (MOX)	SnO_2_, ZnO, WO_3_, In_2_O_3_, TiO_2_, MoO_3_, *etc*.	- Sputtering - Evaporation
Conducting polymers (CP)	Polypirroles, polytiophenes, *etc*.	- Electrochemical - Casting - Spin-coating
Supramolecular materials	Metal-porphyrins, phthalocyanines, *etc*.	- Electrochemical - Solvent casting
Thick-films MOX	SnO_2_, ZnO, WO_3_, In_2_O_3_, TiO_2_, MoO_3_, *etc*.	- High-temperature material processing - Sol-gel
Functional inorganic materials	Metal catalysts (Pt, Pd, Au, Ag, Ru, Ti, W, Ta, Mo, Cu, *etc*.), dopants, *etc*.	- Sputtering - Evaporation
Molecular organic materials	Cavitands, receptors, enzymes, antibodies, proteins, biomolecules, DNA, *etc*.	- Casting - Langmuir-Blodgett
Composites	Fillers in host-matrix	- Langmuir-Blodgett - Chemical routes - PVD techniques
Nanomaterials	*MOX nanostructures*: nanowires, nanotubes, nanorods, nanocrystals, nanoparticles, *etc*. *Carbon nanostructures* : nanotubes, nanowalls, nanofibers, nanoplatelets, *etc*.	- CVD - PVD - Chemical routes

(PVD = Physical Vapor Deposition ; CVD= Chemical Vapor Deposition)

**Table 2.** Transducers used in chemical solid-state sensors.

**Transducer**	**Principle of operation**	**Methods of Fabrication**	**Input/Output**
Conductometric	Electrical Conductivity: • Conducting Polymers • Metal Oxides	PVD Microfabrication MEMS Screen printing	Δc → Δσ → Δi → ΔV
Optical	Absorption; Emission Fluorescence Chemiluminescence Evanescent Wave Fiber Optics	Dip coating MEMS Microfabrication	Δc → Δn → ΔI → Δi →ΔV
Electrochemical	Ionic Conductivity: • Amperometric • Potentiometric • Voltammetric	Screen printing Dip coating MEMS Microfabrication	Δc → Δσ → Δi → ΔV
Thermal	Flow of thermal energy: • Catalytic • Pyroelectric • Calorimetric	PVD Microfabrication	Δc → ΔT → Δi → ΔV
MOSFET	Charge capacitive coupling	Microfabrication	Δc → ΔΦ → Δi → ΔV
Ultrasonic or Mechanical or Acoustic	Piezoelectricity: • QCM • SAW • TFBAR	PVD Screen printing Microfabrication MEMS	Δc → Δm → Δf Δc → Δm → Δf, Δϕ

MEMS = Micro Electro-Mechanical Systems; QCM = Quartz Crystal Microbalance;

SAW = Surface Acoustic Wave; TFBAR = Thin Film Bulk Acoustic Resonator;

Δc = variation of concentration; Δσ = variation of electrical conductivity; Δi = variation of current;

ΔV = variation of voltage; Δn = variation of refractive index; ΔI = variation of light intensity;

ΔT = variation of temperature; ΔΦ = variation of work function; Δm = variation of mass;

Δf = variation of frequency; Δϕ = variation of phase of acoustic wave

**Table 3.** Characteristics of odour measurement techniques.

	
	**Olfactometry**	**Other sensorial methods**	**Electronic Nose**	**GC-O**

Objective measurement of odour concentration	+	+	–	+
Quantitative measurement of odour concentration	+	–	+	+
Measurement standardization	+	+/–	–	–
Continuous measurement	–	+/–	+	–
Single species determination	–	–	–	+
Temporal representativity of measurement	–	+/–	+	–
Time of analysis	+/–	–	+	+/–
Costs	+	+/–	–	+

(+ = high;

+/– = medium;

– = low)
